# Effects of timely case conferencing between general practitioners and specialist palliative care services on symptom burden in patients with advanced chronic disease: results of the cluster-randomised controlled KOPAL trial

**DOI:** 10.1186/s12904-024-01623-z

**Published:** 2024-12-20

**Authors:** Tina Mallon, Josefine Schulze, Nadine Pohontsch, Thomas Asendorf, Jan Weber, Silke Böttcher, Uta Sekanina, Franziska Schade, Nils Schneider, Judith Dams, Michael Freitag, Christiane Müller, Friedemann Nauck, Tim Friede, Martin Scherer, Gabriella Marx

**Affiliations:** 1 Department of General Practice and Primary Care, University Medical Center Hamburg-Eppendorf, Martinistr. 52, 20246 Hamburg, Germany; 2 Department of Medical Statistics, University Medical Center Göttingen, Humboldtallee 32, 37073 Göttingen, Germany; 3 Institute for General Practice and Palliative Care, Hannover Medical School, Carl-Neuberg-Str. 1, 30625 Hannover, Germany; 4 Division of General Practice, Carl von Ossietzky University of Oldenburg, Ammerländer Heerstr. 114-118, 26129 Oldenburg, Germany; 5 Department of General Practice, University Medical Center Göttingen, Humboldtallee 38, 37073 Göttingen, Germany; 6 Department of Palliative Medicine, University Medical Center Göttingen, Von-Siebold-Str. 3, 37075 Göttingen, Germany; 7 Department of Health Economics and Health Care Research, University Medical Center Hamburg-Eppendorf, Martinistr. 52, 20246 Hamburg, Germany

**Keywords:** Palliative Care, Chronic Disease, Family Practice, Primary Health Care, Heart Failure, COPD, Dementia, Pain

## Abstract

**Background:**

Patients with advanced chronic non-malignant conditions often experience significant symptom burden. Therefore, overcoming barriers to interprofessional collaboration between general practitioners (GPs) and specialist palliative home care (SPHC) teams is essential to facilitate the timely integration of palliative care elements. The KOPAL trial aimed to examine the impact of case conferences between GPs and SPHC teams on symptom burden and pain in patients with advanced chronic heart failure, chronic obstructive pulmonary disease, and dementia.

**Methods:**

The cluster-randomised controlled trial compared a structured palliative care nurse visit followed by an interprofessional case conference to usual care. Data were collected from GPs at baseline and 48 weeks, while standardised patient interviews were conducted at baseline, 6, 12, 24, and 48 weeks.

**Results:**

We analysed 172 patients from 49 German GP practices. Both groups showed marginal improvement in symptom burden; however, no statistically significant between-group difference was found ($\:{\Delta\:}$=-0.561, 95% CI: -3.201–2.079, *p* = .68). Patients with dementia experienced a significant pain reduction ($\:{\Delta\:}$=2.187, 95% CI: 0.563–3.812, *p* = .009). Conversely, the intervention did not have a significant effect on pain severity ($\:{\Delta\:}$=-0.711, 95% CI: -1.430 − 0.008, *p*=.053) or pain interference ($\:{\Delta\:}$=-0.036, 95% CI:-0.797 − 0.725, *p*=.926) in other patient groups.

**Conclusions:**

The intervention showed promise in the timely introduction of palliative care elements to address pain management in patients with dementia. Further studies are needed to identify and effectively address symptom burden and pain in other patient groups.

**Trial registration:**

German Clinical Trials Register: https://www.drks.de/DRKS00017795 (Registration date: 9th January 2020).

**Supplementary Information:**

The online version contains supplementary material available at 10.1186/s12904-024-01623-z.

## Background

Progressive chronic non-malignant diseases such as congestive heart failure (CHF), chronic obstructive pulmonary disease (COPD), and advanced dementia are the leading causes of mortality among the ageing population worldwide [1]. In contrast to malignant diseases, they are often characterised by their long duration, slow progression, and prognostic uncertainty [2]. Nonetheless, they cause similar symptoms such as pain, depression, fatigue, and dyspnoea which are equally common in malignant diseases [3]. For example, pain prevalence rates range from 46 to 56% in patients with any type of dementia [4], and up to 66% in patients with moderate to severe COPD [5]. Pain is also prevalent in up to 84% of patients with CHF, often with multiple pain sites [6]. The management of symptoms in chronic non-malignant diseases is complicated by recurring patterns of decompensation and subsequent periods of recovery [7]. Patients suffer from a declining functional status, refractory breathlessness, and recurrent hospitalisations towards the end of life [8, 9]. Despite these facts, referrals to SPHC are rare as research and policy makers are still working on clear guidelines on initiating the transition from active life-prolonging treatment to palliative care for patients with chronic non-malignant diseases [7, 10, 11]. GPs report many other barriers to providing palliative care, including lack of knowledge about palliative care, symptom management, treatment options, psychological aspects and communication strategies [12].

In general, the debate on the initiation of palliative care has revolved around whether timely or early integration of palliative care is appropriate. However, Hui et al. (2022) concluded that the ‘timely palliative care is early palliative care personalised around patients’ needs and delivered at the optimal time and setting’ [13]. They proposed a four-component approach to ensure the timely referral to SPHC for patients with cancer. Among the four components are routine screening of patient’s demands for supportive care, consensual referral criteria specific to the institution, a system for initiating referrals once a patient meets the criteria, and the resources to provide outpatient SPHC. Given the negative impact of the complex symptom burden on patients’ quality of life [8, 10, 14], timely integration of palliative care elements into regular care may benefit all patients with advanced chronic conditions [15].

In Germany, most of the care for people with advanced chronic non-malignant diseases and palliative care needs is provided by GPs [16]. Primary palliative care can be supplemented by SPHC, which, upon prescription by the attending physician, includes a team of specially trained nurses, palliative care physicians, and other professionals as needed. In recent years, SPHC provision in Germany has increased, while the role of primary palliative care has diminished, suggesting that palliative approaches are being shifted away from rather than integrated into standard GP care [17]. However, overall access to palliative care in the last year of life remains insufficient, meeting less than half of the estimated demand [18].

While GPs are generally open to consulting with SPHC teams and working in partnership to provide palliative care, insufficient communication and fragmented care delivery can hinder interprofessional collaboration [16]. Early and systematic introduction of specialised palliative care could facilitate shared care planning, improve collaboration, and ultimately enhance patient outcomes. To explore this potential, we investigated whether case conferences between GPs and SPHC teams can reduce symptom burden and pain for patients with COPD, CHF, and dementia.

## Methods

This is the secondary analysis from data collected within the German multicentre two-arm cluster-randomised controlled KOPAL trial, which aimed to test the effectiveness of a structured SPHC nurse-patient consultation followed by an interprofessional telephone case conference between SPHC nurse, SPHC physician and GP compared to usual care in patients with non-oncological palliative care needs. During the initial SPHC nurse-patient consultation, the SPHC nurse applied the KOPAL conversation guide [19], a structured conversation tool with focus on palliative care needs of patients with advanced chronic diseases. This consultation was originally planned to take place at home. Due to the COVID-19 pandemic, most of the consultations had to be conducted by telephone. The results of the consultation provided the basis for the interprofessional telephone case conference. Initiating a timely initial collaboration between GPs and PC providers to ensure shared care planning by using the combined expertise of GPs and PC providers was the main goal of the interprofessional case conferences. The primary outcome was the reduction of hospital admissions within 48 weeks after baseline. The design of the study and the intervention have been described in detail elsewhere [20]. Endpoints and statistical analysis were defined a priori [21]. The KOPAL study has been approved by the ethics committees of all participating study centres and complies with the ethical standards of the Declaration of Helsinki. KOPAL has been registered with the German Clinical Trials Register (registration no. DRKS00017795) prior to participant recruitment.

### Recruitment

In brief, all SPHC teams of Northern Germany (*N* = 64) were invited to participate. Once a team was enrolled, all GP practices within their area that met the inclusion criteria (specialisation in primary care or internal medicine, focus on primary care medicine, use of computer-based documentation software) were invited for participation. GPs trained as palliative care specialists or currently working in a SPHC team were excluded. A total of 71 practices were randomised to either the intervention or control group utilising a web-based application for block randomisation, incorporating stratification based on the study centre. As patients, providers and researchers were directly involved in the intervention, blinding was not possible.

GPs screened their patients for eligibility. Patients had to meet at least one of the following criteria: (a) confirmed diagnosis of CHF with NYHA class 3–4 [22] and/or (b) COPD with GOLD class 3–4, group D [23] and/or (c) dementia with stage 4 or above in the Global Deterioration Scale [24]. Additionally, the patients were required to have had at least one consultation with the GP within the last three months and needed to provide written and verbal consent. Patients diagnosed with dementia were required to provide verbal informed consent and written consent through a consent form signed by both the patient and their legal representative. Patients diagnosed with cancer in the last five years, currently receiving SPHC support or residing in a care home were excluded. All eligible patients were invited to participate in the study in written form by their GP. Patients were followed up at 6, 12, 24 and 48 weeks after the intervention.

### Data collection

Data collection took place between February 2020 and March 2022. In standardised interviews, patients and, in case of dementia, their family caregivers provided information on sociodemographic characteristics, health status, symptom burden, and palliative care needs. We also assessed number of hospital admissions, days spent in palliative care units, emergency hospital admissions as well as advance directives, preferred place of death and impact of the COVID-19 pandemic on healthcare. Additional clinical information was obtained from the GPs at baseline and 48-week follow-up. The patients’ sociodemographic data included sex, age, education level, marital status, living situation and migration background. Symptom burden and palliative care needs were measured with the Integrated Palliative Care Outcome Scale (IPOS), which is available as a self-report and proxy-report version [25]. The IPOS total score is calculated as the sum of 17 items assessing impairments related to physical symptoms, psychological and emotional problems as well as communication needs during the week prior. The total IPOS score ranges from 0 to 68, with higher scores indicating greater symptom burden. The Brief Pain Inventory (BPI) [26] is a self-report tool specifically designed to evaluate pain intensity and pain interference within a 24-hour recall period. It employs ten-point Likert scales, spanning from 0 (absence of pain or impairment) to 10 (the most severe outcome). Pain intensity is calculated as the mean score derived from four items that capture the extent of worst, least, average, and current pain. Similarly, pain interference is determined by computing the mean of seven items that explore the impact of pain on various aspects of daily functioning. In patients with dementia, we used the Pain Assessment in Advanced Dementia Scale (PAINAD) [27] to assess pain. It is computed as the sum score on five items on observation of breathing, negative vocalisation, facial expression, body language and response to comfort, with a range of 0 to 10 points each. Due to recruitment restrictions following the COVID-19 pandemic, the sample size was reduced from the original planning to 191 participants, resulting in 51 practices with approximately four participants each. This size is designed to detect significant differences in annualised hospitalization rates between intervention and control groups with a statistical power of 80%, assuming a 40% reduction, a two-sided 5% significance level, a 20% dropout rate and an overdispersion assumption of 2 in a Poisson model.

### Statistical analysis

Study participants who completed baseline and at least one follow-up assessment were included in the primary analysis population. Secondary outcomes where compared between groups using linear mixed-effects models [28] adjusted for baseline test scores and number of comorbidities and random intercepts to assess for intra-individual and intra-cluster correlation. Differences at week 48 were calculated using estimated marginal means [29], also referred to as least square means, and 95%-confidence intervals mean differences were reported. Testing was performed at two-sided significance level of α = 0.05. Sensitivity analyses were performed to estimate differences between the analysed sample and the sample obtained by using 10 imputed datasets with multivariate imputations by chained equations [30].

## Results

### Study sample

**Fig. 1:**
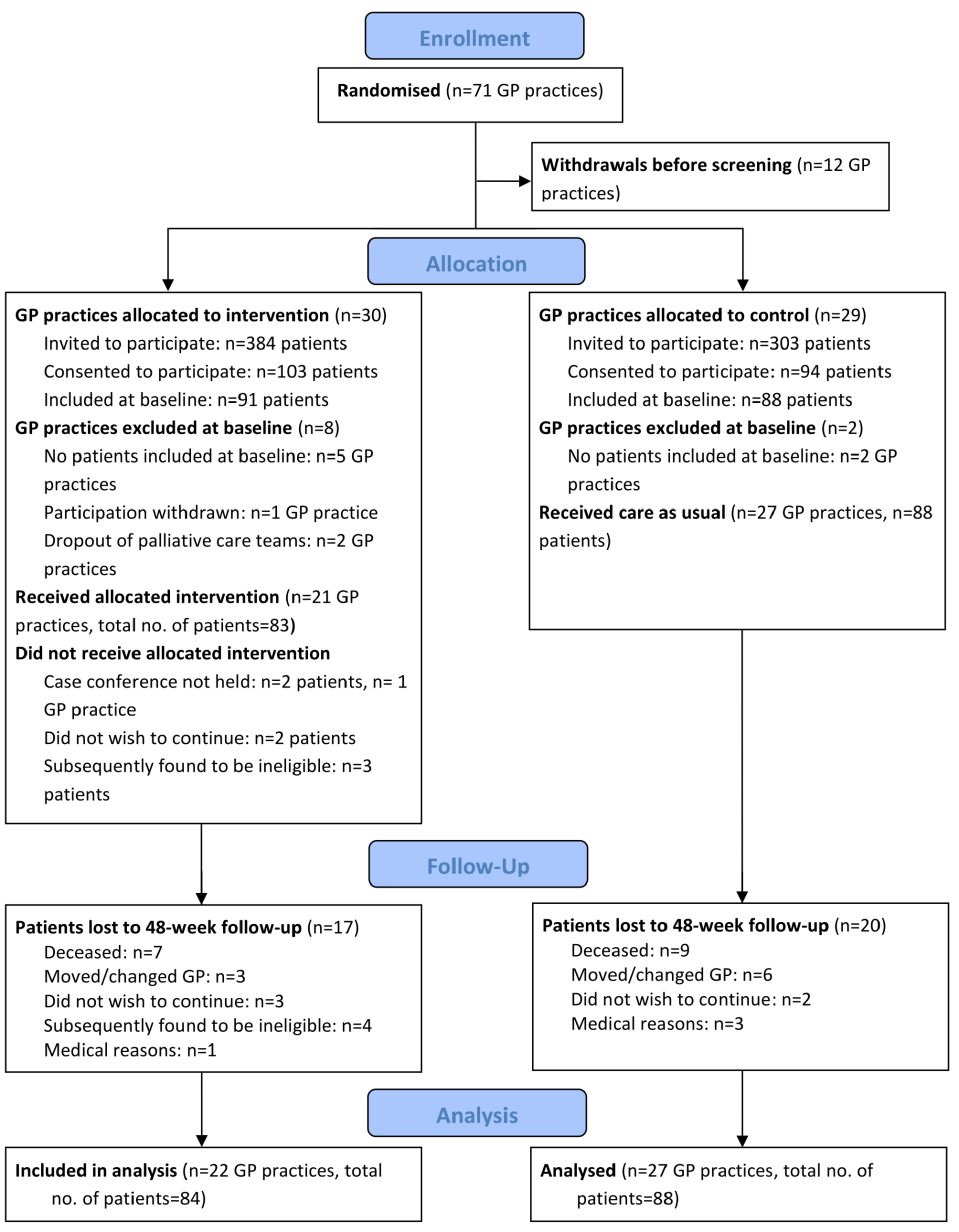
CONSORT flow diagram for the KOPAL cluster-randomised controlled trial

The recruitment process is summarised in Fig. 1. A total of 14 SPHC teams participated in the KOPAL study. Within the areas covered by the teams, 71 GP practices were recruited and randomly assigned to the two study arms. Over the course of the pandemic, twelve practices withdrew from the study before their patient population was screened for eligibility. Ultimately, 49 practices provided patients for the study. Of the 687 potentially eligible patients invited to the study, 197 agreed to participate (response rate 28.7%). At baseline, 91 patients were enrolled in the intervention group and 88 in the control group. In the intervention group, seven patients were subsequently deemed ineligible due to a cancer diagnosis (*n* = 4) or not meeting the advanced stage of the inclusion diagnosis (*n* = 3). Two patients did not receive the intervention but were retained in the sample per intention-to-treat principle, resulting in 84 patients for the intervention group and 88 for the control group used for statistical analysis. The mean cluster size was on average 3.4 patients per practice.

The intervention group comprised slightly younger participants, with a mean age of 75.5 years, as opposed to 77.0 years in the control group. Notably, a greater proportion of men were enrolled in the intervention group (58.3%) than in the control group (51.1%). Although the distribution of inclusion diagnoses was comparable across groups, the prevalence of depression and anxiety was higher in the intervention group (33.3% vs. 18.2% and 21.4% vs. 14.8%, respectively). Moreover, we observed differences in baseline test scores between the intervention and control group, with higher scores in the intervention group for all outcome parameters except PAINAD, where baseline scores were comparable between the two groups (see Appendix). Table 1 provides a detailed description of the study sample.

**Table 1:** Sample characteristics at baseline

Patient characteristics	Intervention (*n* = 84)	Control (*n* = 88)
Mean age, years (SD)	75.5 (9.8)	77.0 (9.9)
Gender, n (%)		
Women Men	35 (41.7) 49 (58.3)	43 (48.9) 45 (51.1)
Living situation, n (%)		
Living alone Living together with children, partner or other people	27 (32.1) 57 (67.9)	33 (37.5)^a^ 54 (61.4)
Marital status, n (%)		
Single Married Divorced Widowed	5 (6.0) 47 (56.0) 10 (11.9) 22 (26.2)	10 (11.4) 50 (56.8) 5 (5.7) 23 (26.1)
Education, n (%)		
No formal education Primary or lower secondary school education Middle school education Technical school certificate High school diploma	1 (1.2) 55 (65.5) 17 (20.2) 6 (7.1) 5 (6.0)	2 (2.3) 55 (62.5) 15 (17.0) 4 (4.6) 12 (13.6)
Diagnosis of inclusion, n (%)		
CHF COPD Dementia	39 (46.4) 33 (39.3) 20 (23.8)	42 (47.7) 35 (39.8) 20 (22.7)
Comorbidities, n (%)		
Depression Anxiety Diabetes Coronary heart disease Renal failure	28 (33.3) 18 (21.4) 26 (31.0) 34 (40.5) 29 (34.5)	16 (18.2) 13 (14.8) 31 (35.2) 27 (30.7) 18 (20.5)
Mean no. of comorbidities (SD)	4.0 (2.0)	3.9 (2.08)
Baseline test scores		
Symptom burden (IPOS), M (SD)	22.9 (10.8)^b^	18.2 (8.7)^c^
Pain severity (BPI), M (SD)	2.8 (2.3)^d^	1.7 (2.0)^e^
Pain interference (BPI), M (SD)	3.3 (2.9)^f^	1.8 (2.3)^g^
Pain Assessment in Advanced Dementia, M (SD)	1.7 (2.2)^h^	1.5 (1.9)^i^

*Notes*: M = mean, SD = standard deviation. ^a^*n* = 87, ^b^*n* = 65, ^c^*n* = 76, ^d^*n*=62, ^e^*n* = 66, ^f^*n* = 61, ^g^*n* = 64, ^h^*n* = 18, ^i^*n* = 19

### Effects of the KOPAL intervention on symptom burden and pain

As shown in Table 2, the intervention did not have a statistically significant effect in lowering symptom burden, pain severity or pain interference scores at week 48 for patients with COPD or CHF (IPOS: $\:{\Delta\:}$ = − 0.561, 95% CI: -3.201 to 2.079, *p* = .676; BPI pain severity: $\:{\Delta\:}$ = − 0.711, 95% CI: -1.430 to 0.008, *p* = .053; BPI pain interference: $\:{\Delta\:}$ = − 0.036, 95% CI: − 0.797 to 0.725, *p* = .926).

**Table 2:** Effects on symptom burden and pain: Estimated marginal mean differences at 48 weeks

Outcome parameter, *N*	Main analysis				Sensitivity analysis (with imputed datasets)			
	$\:{\Delta\:}^{*}$	95% CI		*p*	$\:{\Delta\:}^{*}$	95% CI		*p*
Symptom burden (IPOS score), *N* = 131	− 0.561	-3.201,	2.079	0.676	− 0.341	-2.829,	2.146	0.787
Pain severity (BPI score), *N* = 119	− 0.711	-1.430,	0.008	0.053	− 0.640	-1.362,	0.082	0.082
Pain interference (BPI score), *N* = 115	− 0.036	− 0.797,	0.725	0.926	− 0.249	− 0.947,	0.449	0.484
Pain Assessment in Advanced Dementia (PAINAD score), *N* = 34	**2.187**	0.563,	3.812	0.009	**2.130**	0.577,	3.684	0.008

Notes: ^*^Estimated marginal mean difference at 48 weeks (control-intervention) with 95% confidence intervals (CI). Bold values statistically significant (*p* < .05). IPOS = Integrated Palliative Care Outcome Scale, BPI = Brief Pain Inventory

**Fig. 2:**
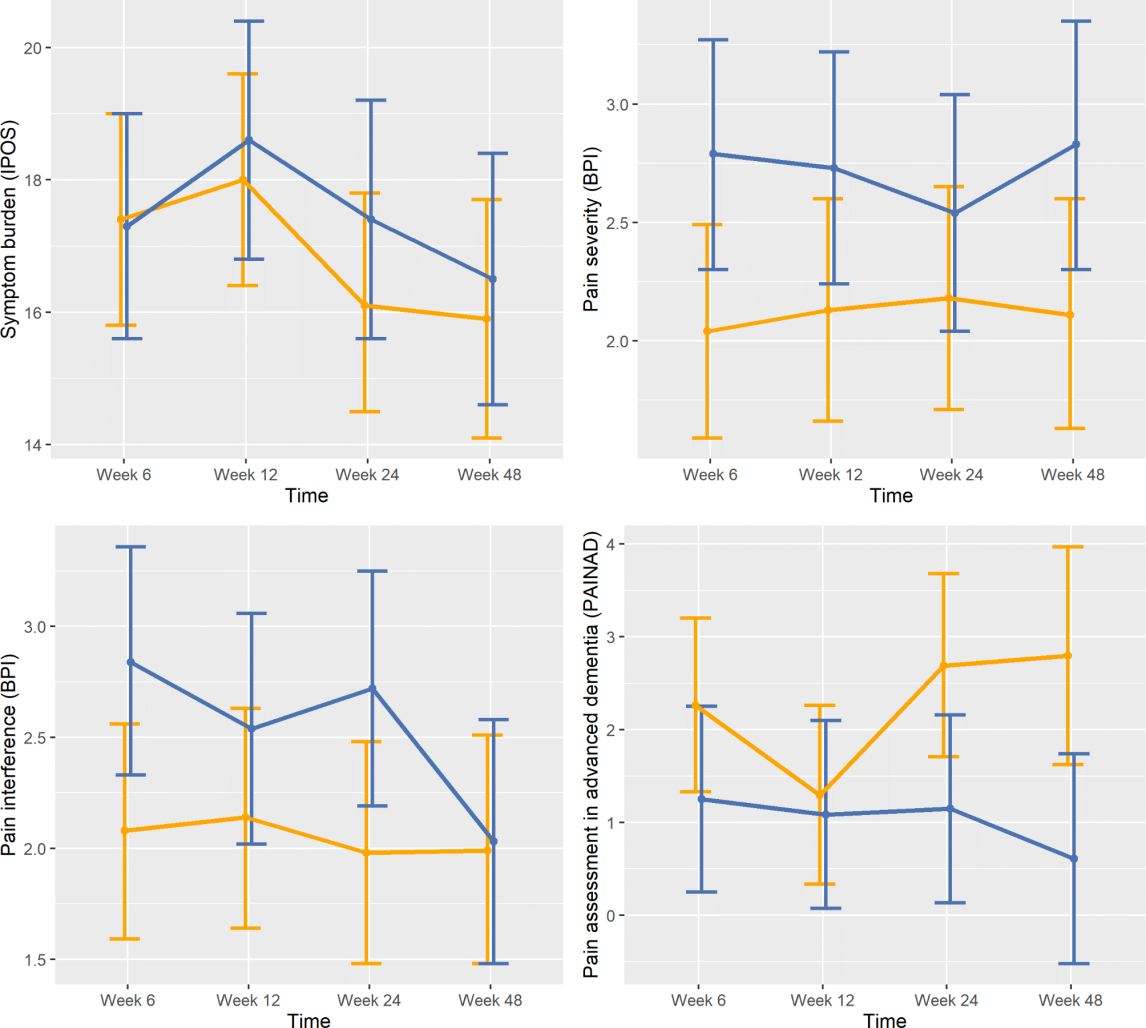
Comparing baseline-adjusted estimated marginal means with 95% confidence intervals by outcome measure and study arm Legend: Intervention group in blue, control group in orange

In the dementia subgroup, the control group had significantly higher pain scores at week 48, as measured by PAINAD ($\:{\Delta\:}$ = 2.187, 95% CI: 0.563 to 3.812, *p* = .009). Figure 2 visualises the differences of baseline-adjusted least square means between intervention and control group over time.

## Discussion

### Main findings

In the longitudinal analyses of the KOPAL study, we investigated whether symptom burden and pain in patients with advanced COPD, CHF and dementia could be reduced by implementing timely case conferences between GPs and SPHC teams. Neither the IPOS scale, which measures symptom burden, nor the BPI, which measures pain intensity and pain interference, showed a significant improvement in the intervention group. We did, however, find a significant reduction in pain in patients with dementia in the intervention group at 48 weeks. This finding is particularly important as pain is often underdiagnosed and undertreated in people with dementia [31–33]. Informal carers and nurses face significant challenges in recognising and evaluating pain in people with dementia due to the complexity of pain assessment, difficulties in differentiating symptoms of dementia from signs of pain, lack of interprofessional collaboration, and time constraints [34]. This highlights the need for specific training of nurses in the field of pain management in people with dementia. Our intervention may have addressed this gap by using a standardised pain assessment through an SPHC nurse and discussing the findings in the case conferences.

### Strengths and limitations

This study is, to the best of our knowledge, the first to investigate the effects of timely case conferencing on pain and symptom burden between GPs and SPHC for patients with advanced chronic non-malignant diseases in Germany. As the number of patients with chronic conditions is rising, our findings are valuable to researchers and policymakers in guiding future courses of action. During the conduct of the study, we encountered several challenges. The COVID-19 pandemic posed a significant obstacle, leading to deviations from our original study design and challenges in recruiting participants within the allocated funding period. As a result, recruitment had to be halted, leading to higher statistical uncertainty of our results. Furthermore, the limited sample size of the dementia subgroup increases the susceptibility of our results to bias and the influence of symptom variability. A detailed description of the pandemic’s impact on the KOPAL study can be found elsewhere [16]. Irrespective of the pandemic, we observed imbalances in our sample due to cluster randomisation at the practice level. Specifically, the intervention group had higher baseline levels of symptom burden and a higher prevalence of depression and anxiety. Due to logistical constraints, we were unable to stratify randomisation by baseline test scores. This would have required the simultaneous assessment of a large number of patients, and the high caseload of the SPHC teams across multiple GP practices made it impractical to complete the intervention procedures shortly after baseline. This approach was necessary to minimise potential changes in test scores, given the frequent deterioration and hospitalisation of this patient population. As a result, we cannot rule out the possibility of selection bias. Another challenge was the identification of suitable participants due to inconsistent documentation of disease severity in GP practices, which often meant that severity had to be established from clinical data to ensure the accuracy of the sample.

### Comparison with existing literature

Our findings are in line with earlier research that demonstrated limited benefits of single case conferences on clinical patient outcomes [35]. Although the intervention did not have a significant impact on symptom burden and pain in participants with COPD and CHF, this study illustrates the need for further research to address the suboptimal end-of-life care for patients with non-malignant chronic diseases [36].

Our study population had only a slightly lower overall symptom burden than patients already receiving palliative care, compared with the sample used to validate the outcome measure (20.1 vs. 27.4 points) [25]. This emphasises that KOPAL seems to have targeted the right patient groups even though no significant results could be shown for patients with COPD and CHF. However, a recent review on the integration of palliative care in COPD highlights that SPHC expertise can be used well before end-stage COPD and should be integrated early to realise its potential to provide significant benefits on patient-reported outcomes and end of life [9, 37].

Also, the findings from our longitudinal analysis indicate a trend towards a reduction in symptom burden in the overall sample. This may point the presence of non-specific factors that affected the outcome regardless of group allocation, such as interviewer qualities, participant motivation, or the relationship between the two parties [38]. In our study, the repeated interviewer-participant contact over the five interview time points may have had a beneficial effect on the symptom burden outcome and may hint at an unmet need for psychosocial support.

It remains to be investigated whether the effects of the case conference may surface at a later stage during the patient’s course of the disease as arrangements made during case conferences may only come into play when the patient’s condition deteriorates significantly. This suggests that palliative care approaches tailored to the needs of patients with advanced chronic non-malignant diseases, as well as assessment tools to determine the optimal time of transition to palliative care, may be necessary to initiate SPHC at an appropriate time and ensure the most effective treatment and support.

## Conclusions

Interprofessional case conferences between GPs and SPHC teams provide an opportunity for healthcare providers to share information, discuss treatment options, and develop individualised care plans for patients with progressive chronic non-malignant diseases. The results of our study suggest that implementing timely case conferences to introduce elements of palliative care can improve pain management in patients with dementia, highlighting the crucial role of SPHC nurses in this process and emphasising the need for additional training in pain management for this patient population. Future research should examine whether the positive effects observed in the dementia subgroup can be replicated, and explore the mechanisms underlying these outcomes. Although the KOPAL intervention shows promise, further studies are necessary to identify potential barriers to effectively managing symptom burden and pain in patients with COPD and CHF. Our study provides insights into the potential benefits of timely case conferences and underscores the need for continued efforts to address palliative care needs of patients with chronic non-malignant diseases.

## Electronic Supplementary Material

Below is the link to the electronic supplementary material.

Supplementary Material 1

## Data Availability

De-identified data will be available upon reasonable request ending 7 years after this publication. Proposals should be sent to Gabriella Marx (g.marx@uke).

## Abbreviations

CHF: Congestive heart failure
COPD: Chronic obstructive pulmonary disease
GP: General practitioner
SPHC: Specialist palliative home care
IPOS: Integrated Palliative Care Outcome Scale
BPI: Brief Pain Inventory
PAINAD: Pain Assessment in Advanced Dementia Scale
M: Mean
SD: Standard deviation
CI: Confidence interval
