# Supplementary material for: Effects of timely case conferencing between general practitioners and specialist palliative care services on symptom burden in patients with advanced chronic disease: results of the cluster-randomised controlled KOPAL trial
Source: BMC Palliat Care. 2024 Dec 20;23:293. doi: 10.1186/s12904-024-01623-z (PMC11662516; doi:10.1186/s12904-024-01623-z)
Supplement: Supplementary file 1 — Supplementary Material 1 [file 12904_2024_1623_MOESM1_ESM.docx]

Appendix: Descriptive PAINAD score distribution (mean ± standard error) for dementia subgroup participants in the control and intervention groups at baseline and follow-up


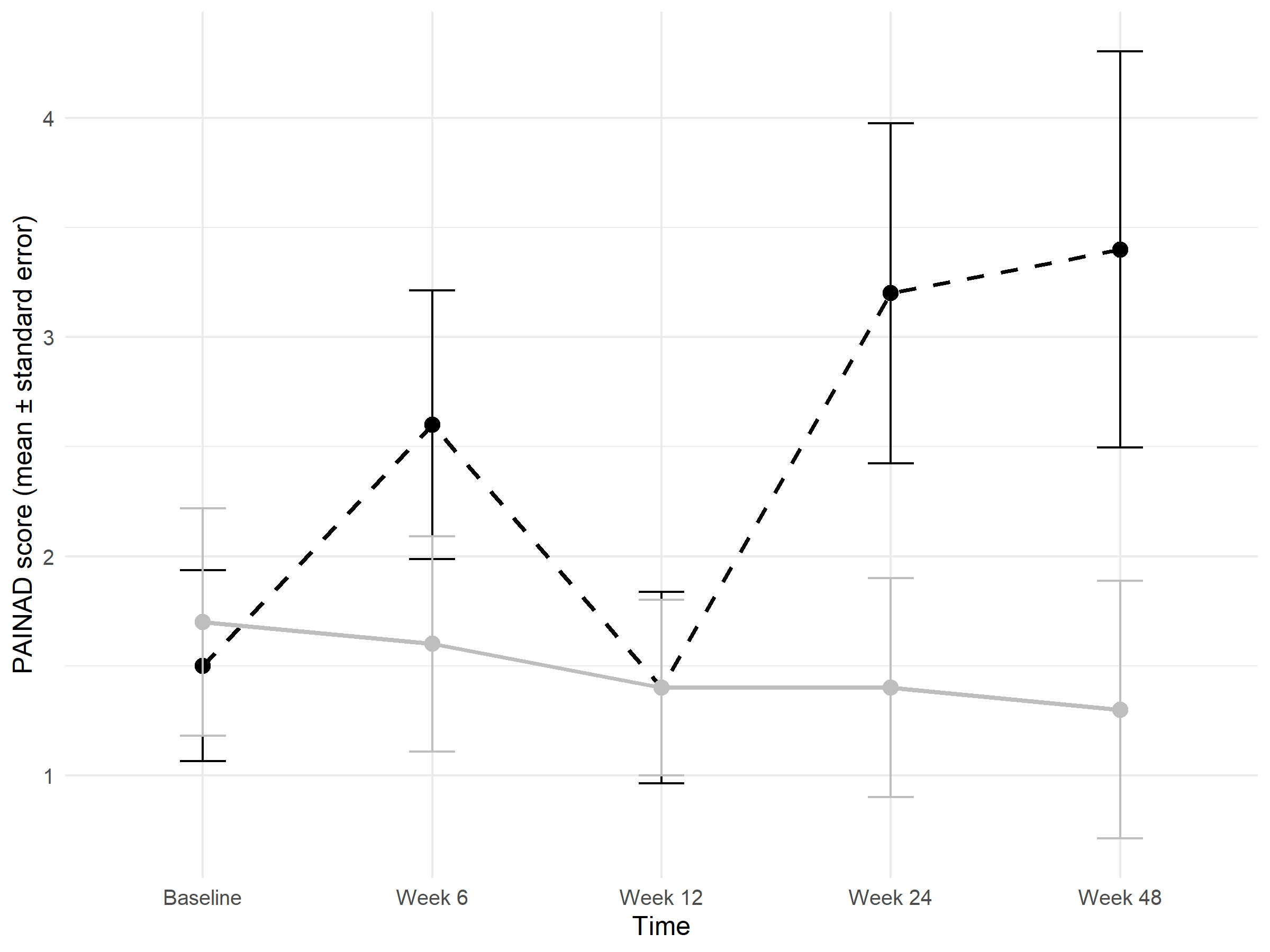


*Notes*: Intervention group is represented by a solid grey line, control group is represented by a dashed black line.
